# USE OF NEW HEPATITIS C DRUGS IN MEDICARE: PERSISTENT URBAN-RURAL DISPARITIES AND POTENTIAL INTERVENTIONS

**DOI:** 10.1093/geroni/igz038.047

**Published:** 2019-11-08

**Authors:** Ping Du

**Affiliations:** Pennsylvania State University College of Medicine, Hershey, Pennsylvania, United States

## Abstract

Chronic hepatitis C virus (HCV) infection, whose prevalence is concentrated among older adults, can bring serious health impacts and high financial burden. With the availability of highly effective and well tolerated direct-acting antiviral (DAA) therapy, treatment of chronic HCV infection has rapidly evolved, making HCV treatment less burdensome. However, the high cost of DAA and lack of clinical expertise are still important barriers for providing DAA therapy to rural patients, highlighting the urban-rural disparities in managing many chronic diseases for aging populations. Telehealth could serve as effective care-model to improve management of HCV infection for older, rural populations. This talk will present work that examines multi-level factors affecting HCV DAA treatment (focusing on urban-rural disparities), evaluates changes in urban-rural disparities in DAA utilization over time, and explores the role of a telehealth-based intervention in reducing urban-rural disparities in HCV DAA treatment in Medicare patients.

